# Supplementary material for: Naldemedine is associated with earlier defecation in critically ill patients with opioid-induced constipation: A retrospective, single-center cohort study
Source: PLoS One. 2024 Jan 3;19(1):e0295952. doi: 10.1371/journal.pone.0295952 (PMC10763934; doi:10.1371/journal.pone.0295952)
Supplement: S1 Table — (DOCX) [file pone.0295952.s001.docx]

**S1 Table. Patient characteristics stratified by defecation status**

|  | All | Defecation | No Defecation | P value |
| --- | --- | --- | --- | --- |
| Sample size | 875 | 433 | 442 |  |
| Age [median (IQR)] | 69 [56–76] | 70 [58–76] | 67 [53–75] | 0.002 |
| Male gender [N (%)] | 596 (68.1) | 295 (68.1) | 301 (68.1) | 1.000 |
| BMI (kg/m^2^) [median (IQR)] | 23.0 [20.1–26.0] | 22.9 [19.6–25. 8] | 23.3 [20.6–26.3] | 0.014 |
| Maintenance hemodialysis | 51 (5.8) | 29 (6.7) | 22 (5.0) | 0.31 |
| Reason for ICU admission [N (%)] |  |  |  | <0.001 |
| Medical (non-operative) | 398 (45.5) | 236 (54.5) | 162 (36.7) |  |
| Surgical—elective (operative) | 211 (24.1) | 76 (17.6) | 135 (30.5) |  |
| Surgical—emergency (operative) | 266 (30.4) | 121 (27.9) | 145 (32.8) |  |
| Main disease category [N (%)] |  |  |  | 0.010 |
| Cardiovascular | 395 (45.1) | 176 (40.6) | 219 (49.5) |  |
| Respiratory | 207 (23.7) | 123 (28.4) | 84 (19.0) |  |
| Neurological | 92 (10.5) | 45 (10.4) | 47 (10.6) |  |
| Abdominal | 60 (6.9) | 26 (6.0) | 34 (7.7) |  |
| Others | 121 (13.8) | 63 (14.5) | 58 (13.1) |  |
| APACHE II score [median (IQR)] | 20 [15–25] | 21 [16–27] | 19 [14–24] | <0.001 |
| Type of opioid [N (%)] |  |  |  |  |
| Fentanyl | 871 (99.5) | 433 (100.0) | 438 (99.1) | 0.12 |
| Morphine | 5 (0.6) | 0 (0.0) | 5 (1.1) | 0.062 |
| Mechanical ventilation [N (%)] | 808 (92.3) | 405 (93.5) | 403 (91.2) | 0.21 |
| Receiving other sedatives [N (%)] | 410 (46.9) | 205 (47.3) | 205 (46.4) | 0.79 |
| Receiving vasoactive drugs [N (%)] | 258 (29.5) | 123 (28.4) | 135 (30.5) | 0.51 |
| Receiving muscle relaxants [N (%)] | 30 (3.4) | 19 (4.4) | 11 (2.5) | 0.14 |
| PaO2/FiO2 (mmHg)[median (IQR)] | 240 [175–308] | 232 [170–303] | 249 [181–315] | 0.068 |
| Creatinine (mg/dL) [median (IQR)] | 0.97 [0.66–1.78] | 1.02 [0.67–1.92] | 0.92 [0.65–1.59] | 0.15 |
| Bilirubin (mg/dL) [median (IQR)] | 0.73 [0.46–1.25] | 0.69 [0.44–1.27] | 0.76 [0.47–1.22] | 0.13 |
| RASS [median (IQR)] | -1 [-3– -1] | -1 [-3– -1] | -1 [-3– -1] | 0.85 |
| Antibiotics use [N (%)] | 340 (38.9) | 155 (35.8) | 185 (41.9) | 0.071 |
| Enteral nutrition use [N (%)] | 431 (49.3) | 246 (56.8) | 185 (41.9) | <0.001 |
| Laxative at inclusion [N (%)] | 37 (4.2) | 26 (6.0) | 11 (2.5) | 0.011 |
| Erythromycin use [N (%)] | 2 (0.2) | 2 (0.5) | 0 (0.0) | 0.25 |
| Metoclopramide use [N (%)] | 42 (4.8) | 20 (4.6) | 22 (5.0) | 0.88 |

BMI: body mass index, GI: Gastrointestinal, APACHE II: acute physiology and chronic health evaluation II, RASS: Richmond agitation sedation score. IQR: Interquartile range.
