# Supplementary material for: Naldemedine is associated with earlier defecation in critically ill patients with opioid-induced constipation: A retrospective, single-center cohort study
Source: PLoS One. 2024 Jan 3;19(1):e0295952. doi: 10.1371/journal.pone.0295952 (PMC10763934; doi:10.1371/journal.pone.0295952)
Supplement: S2 Table — (DOCX) [file pone.0295952.s002.docx]

**S2 Table. Rescue laxatives received during the study**

|  | All | Naldemedine | No Naldemedine | P value |
| --- | --- | --- | --- | --- |
| Number of patients | 875 | 63 | 812 |  |
| Type of rescue laxatives [n (%)] |  |  |  |  |
| Picosulfate | 80 (9.1) | 12 (19.0) | 68 (8.4) | 0.010 |
| Magnesium oxide | 56 (6.4) | 9 (14.3) | 47 (5.8) | 0.015 |
| Lubiprostone | 14 (1.6) | 1 (1.6) | 13 (1.6) | 1.000 |
| Sennoside | 11 (1.3) | 3 (4.8) | 8 (1.0) | 0.039 |
| Lactulose | 1 (0.1) | 1 (1.6) | 0 (0.0) | 0.072 |
