# Supplementary material for: Naldemedine is associated with earlier defecation in critically ill patients with opioid-induced constipation: A retrospective, single-center cohort study
Source: PLoS One. 2024 Jan 3;19(1):e0295952. doi: 10.1371/journal.pone.0295952 (PMC10763934; doi:10.1371/journal.pone.0295952)
Supplement: S1 File — (DOCX) [file pone.0295952.s004.docx]

Variables

age: years old

gender: 0 (female) 1(male)

bmi: kg/m^2^

admcateg: admission categories, 1(non-operative) 2(post elective surgeries) 3(post emergency surgeries)

main_dis_cat: main disease categories, 1(cardiovascular) 2(respiratory) 3(neurological) 4(abdominal) 5(others)

rass: Richmond agitation sedation score

apache2score: APACHE II score

maintdialysis: undergoing maintenance dialysis, 0(no) 1(yes)

fentanyl: fentanyl use at the time of inclusion, 0(no) 1(yes)

morphine: morphine use at the time of inclusion, 0(no) 1(yes)

muscle_relaxants: receiving muscle relaxants at the time of inclusion, 0(no) 1(yes)

vasoactive_drugs: receiving vasoactive drugs at the time of inclusion, 0(no) 1(yes)

other_sedatives: receiving other sedatives at the time of inclusion, 0(no) 1(yes)

en_use: enteral nutrition use at the time of inclusion

abx_use: antibiotics use at the time of inclusion

metoclopramide: metoclopramide use at the time of inclusion

laxat_Inc: receiving laxative at the time of inclusion, 0(no) 1(yes)

bilirubin: bilirubin (mg/dL) at the time of inclusion or within 24 hours prior

creatinine: creatinine (mg/dL) at the time of inclusion or within 24 hours prior

PF_ratio: PaO2/FiO2 (mmHg) at the time of inclusion or within 24 hours prior

mv_inclusion: mechanical ventilator use at the time of inclusion, 0(no) 1(yes)

totalmv: the length of the mechanical ventilation, days

nald_start: time to the administration of naldemedine from inclusion, hours

rescue_start: time to the administration of rescue laxative from inclusion, hours

rescue_detail: the name of the prescribed rescue laxative

defecation: the time of the defecation, hours

diarrhea: diarrhea during the observation period, 0(no) 1(yes)

icudisch_inc: the time of the ICU discharge from inclusion, hours

observation: observation period, hours

los_icu: the length of the ICU stay, days

hospideath: death at the time of hospital discharge, 0(no) 1(yes)

icudeath: death at the time of ICU discharge, 0(no) 1(yes)

icudisch_on_mv: mechanical ventilation at the time of ICU discharge, 0(no) 1(yes)

icu_death_on_mv: ICU death while on mechanical ventilation, 0(no) 1(yes)
