# Supplementary material for: Naldemedine is associated with earlier defecation in critically ill patients with opioid-induced constipation: A retrospective, single-center cohort study
Source: PLoS One. 2024 Jan 3;19(1):e0295952. doi: 10.1371/journal.pone.0295952 (PMC10763934; doi:10.1371/journal.pone.0295952)
Supplement: Attachment — Submitted filename: Response to Reviewers.docx [file pone.0295952.s005.docx]

October 23rd, 2023

Dr. Guilherme Antonio Moreira de Barros

Academic Editor

PLOS ONE

RE: Response to reviewers,

Dear Dr. Moreira de Barros

Thank you for your ongoing consideration of our manuscript for publication in PLOS ONE. We appreciate the time spent by you and the reviewers and believe the revised manuscript is improved. Below, we have addressed your and the reviewers’ comments.

We look forward to your editorial decision.

Sincerely,

Seiya Nishiyama, MD

Tomoyuki Masuyama, MD, PhD

Department of Anesthesiology and Critical Care Medicine, Jichi Medical University, Saitama Medical Center

**EDITOR’S SPECIFIC COMMENTS:**

**1. Please ensure that your manuscript meets PLOS ONE's style requirements, including those for file naming. The PLOS ONE style templates can be found at**

**https://journals.plos.org/plosone/s/file?id=wjVg/PLOSOne_formatting_sample_main_body.pdf and**

**https://journals.plos.org/plosone/s/file?id=ba62/PLOSOne_formatting_sample_title_authors_affiliations.pdf**

[Response]

Thank you for your feedback. We have revised the manuscript to align with the style of your journal. We have added author contributions to the first page.

**2. Thank you for stating the following financial disclosure:**

**“NO”**

**At this time, please address the following queries:**

**a) Please clarify the sources of funding (financial or material support) for your study. List the grants or organizations that supported your study, including funding received from your institution.**

**b) State what role the funders took in the study. If the funders had no role in your study, please state: “The funders had no role in study design, data collection and analysis, decision to publish, or preparation of the manuscript.”**

**c) If any authors received a salary from any of your funders, please state which authors and which funders.**

**d) If you did not receive any funding for this study, please state: “The authors received no specific funding for this work.”**

**Please include your amended statements within your cover letter; we will change the online submission form on your behalf.**

[Response]

As suggested, we have incorporated the recommended text on page 18: “Funding: The authors received no specific funding for this work.”, “Competing interests: The authors have declared that no competing interests exist.”

**3. We note that you have stated that you will provide repository information for your data at acceptance. Should your manuscript be accepted for publication, we will hold it until you provide the relevant accession numbers or DOIs necessary to access your data. If you wish to make changes to your Data Availability statement, please describe these changes in your cover letter and we will update your Data Availability statement to reflect the information you provide.**

[Response]

Thank you for pointing this out. For the purpose of study reproducibility, we will provide the dataset used in this study. We would like to provide the dataset as "S1 Data" and a variable description of the data as "S1 File”. We have added this information to the Supporting information section on page 24 of the manuscript.

**4. Please review your reference list to ensure that it is complete and correct. If you have cited papers that have been retracted, please include the rationale for doing so in the manuscript text, or remove these references and replace them with relevant current references. Any changes to the reference list should be mentioned in the rebuttal letter that accompanies your revised manuscript. If you need to cite a retracted article, indicate the article’s retracted status in the References list and also include a citation and full reference for the retraction notice.**

[Response]

Thank you for pointing this out. We have verified that the reference is complete and correct.

**REVIEWERS' COMMENTS:**

**Reviewer #1:**

**1. The indication and administration details of “rescue laxatives” in this study should be stated.**

[Response]

We have added the following statement to the 'Material and Methods’ section (page6, paragraph 1): 'Based on bowel movement records, rescue laxatives were prescribed at the discretion of the critical care physicians.'

**2. More patients in Naldemedine group received “rescue laxatives”. Is that the reason that more patients defecated in naldemedine group? How many patients in the naldemedine group defecated without using “rescue laxatives”? While how many patients in the no-naldemedine group defecated without using “rescue laxatives”? Is there any statistical difference?**

[Response]

We appreciate these helpful suggestions. After reviewing the data you pointed out, we realized that our previous analysis was partially incorrect. We incorrectly considered patients who were exposed to naldemedine or rescue laxatives after the outcome occurred as 'exposed'. We have provided corrected Data set to reach the conclusion (S1 Data).

Based on your suggestions, we were able to recognize and correct the errors. We performed the analysis again and found that there were 63 patients in ‘Naldemedine group’ and 812 in ‘No naldemedine group'. Similarly, the use of rescue laxative decreased in each group. We analyzed how often patients defecated without rescue laxatives and found no statistically significant difference (p=0.232). We have added this result to Table 2 on page 11 as 'Defecation without rescue laxatives [n (%)]'.

It is important that these analyses are univariate, unadjusted for confounding, and do not affect the results of the main analysis, the Cox proportional hazard regression analysis with time-dependent covariates.

Based on the revised number of participants in each group, we have revised Table 1 (page 10) and Fig. 1. We also revised the Results section (paragraphs 1-3 [pages 8-12]) and Discussion section (paragraphs 1-2 [pages 13-14] and 4 [page 15]), respectively.

**Reviewer #2: Well written presentation of the study conducted by the authors. Good structured manuscript with detailed methodology and results-presenting section. Clearly noted limitation part. And though the overall control group was relatively small (68 patients), I feel that the present paper could be published in its present form.**

[Response]

We thank the reviewer for these excellent comments. As answered above, the number of participants in ‘Naldemedine group’ decreased slightly. However, this change did not affect the results of the main analysis with time-dependent covariates.

**Other minor revisions：**

[1] We have changed the 'N' indicating the number of participants in each table to 'n.' This is more suitable for representing the sample size rather than the population.

[2] We have removed the phrase ‘for secondary outcomes’ from the first paragraph of "Statistical methods" section (page 7).

[3] In Table2 (page 11) 'Death while on MV' has been corrected as 'ICU death while on MV'.

[4] The same words ('study') were duplicated in the first paragraph of 'Strengths and limitations' (page 17), so one has been removed.
